# Preterm birth and the risk of chronic disease multimorbidity in adolescence and early adulthood: A population-based cohort study

**DOI:** 10.1371/journal.pone.0261952

**Published:** 2021-12-31

**Authors:** Katriina Heikkilä, Anna Pulakka, Johanna Metsälä, Suvi Alenius, Petteri Hovi, Mika Gissler, Sven Sandin, Eero Kajantie

**Affiliations:** 1 Population Health Unit, Finnish Institute for Health and Welfare, Helsinki, Finland; 2 Children’s Hospital, University of Helsinki and Helsinki University Hospital, Helsinki, Finland; 3 Information Services Department, Finnish Institute for Health and Welfare, Helsinki, Finland; 4 Academic Primary Health Care Centre, Region Stockholm, Stockholm, Sweden; 5 Department of Molecular Medicine and Surgery, Karolinska Institutet, Stockholm, Sweden; 6 Department of Medical Epidemiology and Biostatistics, Karolinska Institutet, Stockholm, Sweden; 7 Department of Psychiatry, Icahn School of Medicine at Mount Sinai, New York, NY, United States of America; 8 Seaver Autism Center for Research and Treatment at Mount Sinai, New York, NY, United States of America; 9 PEDEGO Research Unit, MRC Oulu, Oulu University Hospital and University of Oulu, Oulu, Finland; 10 Department of Clinical and Molecular Medicine, Norwegian University of Science and Technology, Trondheim, Norway; University of Copenhagen: Kobenhavns Universitet, DENMARK

## Abstract

**Background:**

People who were born prematurely have high risks of many individual diseases and conditions in the early part of the life course. However, our knowledge of the burden of multiple diseases (multimorbidity) among prematurely born individuals is limited. We aimed to investigate the risk and patterns of chronic disease multimorbidity in adolescence and early adulthood among individuals born across the spectrum of gestational ages, comparing preterm and full-term born individuals.

**Methods and findings:**

We used individual-level data from linked nationwide registers to examine the associations of gestational age at birth with specialised healthcare records of ≥2 chronic diseases (multimorbidity) in adolescence (age 10–17 years) and early adulthood (age 18–30 years). Our study population comprised 951,116 individuals (50.2% females) born alive in Finland between 1^st^ January 1987 and 31^st^ December 2006, inclusive. All individuals were followed from age 10 years to the onset of multimorbidity, emigration, death, or 31 December 2016 (up to age 30 years). We estimated hazard ratios (HRs) and 95% confidence intervals (CIs) for multimorbidity using flexible parametric survival models. During 6,417,903 person-years at risk (median follow-up: 7.9 years), 11,919 individuals (1.3%) had multimorbidity in adolescence (18.6 per 10,000 person-years). During 3,967,419 person-years at risk (median follow-up: 6.2 years), 15,664 individuals (1.7%) had multimorbidity in early adulthood (39.5 per 10,000 person-years). Adjusted HRs for adolescent multimorbidity, comparing preterm to full-term born individuals, were 1.29 (95% CI: 1.22 to 1.36) and 1.26 (95% CI: 1.18 to 1.35) in females and males, respectively. The associations of preterm birth with early adult multimorbidity were less marked, with the adjusted HRs indicating 1.18-fold risk in females (95% CI: 1.12 to 1.24) and 1.10-fold risk in males (95% CI: 1.04 to 1.17). We observed a consistent dose-response relationship between earlier gestational age at birth and increasing risks of both multimorbidity outcomes. Compared to full-term born males, those born at 37–38 weeks (early term) had a 1.06-fold risk of multimorbidity in adolescence (95% CI: 0.98 to 1.14) and this risk increased in a graded manner up to 6.85-fold (95% CI: 5.39 to 8.71) in those born at 23–27 weeks (extremely premature), independently of covariates. Among females, the same risks ranged from 1.16-fold (95% CI: 1.09 to 1.23) among those born at 37–38 weeks to 5.65-fold (95% CI: 4.45 to 7.18) among those born at 23–27 weeks. The corresponding risks of early adult multimorbidity were similar in direction but less marked in magnitude, with little difference in risks between males and females born at 36–37 weeks but up to 3-fold risks observed among those born at 23–27 weeks.

**Conclusions:**

Our findings indicate that an earlier gestational age at birth is associated with increased risks of chronic disease multimorbidity in the early part of the life course. There are currently no clinical guidelines for follow-up of prematurely born individuals beyond childhood, but these observations suggest that information on gestational age would be a useful characteristic to include in a medical history when assessing the risk of multiple chronic diseases in adolescent and young adult patients.

## Introduction

Multimorbidity, the co-occurrence of two or more chronic, non-communicable diseases in one individual, is a growing health concern worldwide [1–5]. Multimorbidity is associated with many adverse health outcomes (including. increased mortality and numbers of hospital admissions, and decreased quality of life [6–8] and it incurs considerable human and healthcare costs in all age groups. [9] Many risk factors for multimorbidity (e.g. age, tobacco smoking and socioeconomic deprivation) are now well-recognised, but other potential risk factors and high-risk groups are less well understood. [2, 3, 10–12] One potential risk factor for multimorbidity is preterm birth (being born before 37 completed weeks of gestation) [13, 14].

Preterm birth constitutes approximately 6% of all births in Finland and the Nordic countries, and 11% of births worldwide [13, 15, 16]. Children and adults who were born prematurely have increased risks of many diseases and health conditions, including low cognitive abilities [17, 18] and cardiometabolic [19–25], respiratory [26, 27] and neuropsychiatric diseases [28–30]. Compared to term-born young adults, those who were born preterm have been estimated to have (depending on the degree of prematurity) 2.5- to 4.0-fold increased risk of metabolic syndrome [23, 24], 1.19- to 1.53-fold increased risk of ischaemic heart disease [19], 1.7- to 3.6-fold increased risk of asthma [27], 1.4- to 3.0-fold increased risk of depression [31], 1.35- to 2.31.fold increased risk of autism-spectrum disorders [29] and 1.23- to 1.44-fold increased risk of mortality [32]. However, the association of preterm birth with chronic disease multimorbidity (the co-occurrence of multiple chronic diseases in one individual) is unclear.

### Aims

The primary aim of our study was to investigate the risks of chronic disease multimorbidity in adolescence (age 10–17 years) and young adults (age 18–30 years) across the spectrum of gestational ages, comparing preterm-born individuals and those born at full-term. The secondary aim was to examine the overlap of chronic disease multimorbidity in adolescence and early adulthood with intellectual disabilities and developmental disorders, comparing preterm and full-term born individuals.

## Methods

### Data sources

Data on gestational age at birth, sex, date of birth, birth weight, multiple pregnancy and mother’s characteristics (parity, smoking during pregnancy, socioeconomic position) were ascertained from the Medical Birth Register. Congenital anomalies were ascertained from Register of Congenital Malformations and mother’s diabetes or hypertensive disorder from a combination of data from the Medical Birth Register and Care Register for Health Care. Chronic disease multimorbidity in adolescence and early adulthood, as well as intellectual disabilities and disorders of psychological development, behaviour or personality, were ascertained from the Care Register for Health Care. Information on dates of death and emigration from Finland was obtained from the nationwide Population Information System. All these data sources cover the whole population of Finland for the study period (between 1^st^ January 1987 and 31^st^ December 2016).

### Ethical approval

In Finland, research based solely on analysing existing register data does not require ethical approval or consent from the individuals whose pseudonymised data will be used. The research reported here was approved by the institutional ethics review board of the Finnish Institute for Health and Welfare (THL/1984/6.02.01/2018) and the relevant register authorities. All register data were pseudonymised and linked prior to analysis by Statistics Finland and Information Services, Finnish Institute for Health and Welfare, and the researchers only had access to pseudonymised data.

### Patient and public involvement

Our investigation was based on analysis of existing register data and no patients or members of the public were involved in the design, conduct or interpretation of the study. Due to data security and privacy constraints, it was not possible to involve members of the public in the investigation.

### Study population

The study population comprised all individuals who were born alive in Finland between 1^st^ January 1987 and 31^st^ December 2006 (inclusive) and who remained alive and living in Finland at the age 10 years (n = 953,658). Individuals were followed up from age 10 years to the onset of multimorbidity (in adolescence, age 10–17 years, or in early adulthood, age 18–30 years), death, emigration or 31^st^ December 2016. We excluded individuals with a gestational age earlier than 23 weeks (n = 411), those with implausible data on birth weight alone or birth weight in combination with gestational age (birth weight z-score smaller than -6, birth weight less than 300 grams, or gestational age less than 37 weeks and birth weight z-score larger than 4; n = 252). Further excluded were individuals of undetermined sex (n = 26) and individuals with incomplete data on birth weight and/or gestational age (n = 1,853, 0.2%).

### Exposure

Gestational age in completed weeks was analysed as a categorical variable as follows: 23–27 weeks (extremely preterm), 28–31 weeks (very preterm), 32–33 weeks (moderately preterm), 34–36 weeks (late preterm), 37–38 weeks (early term), 39–41 weeks (full-term, reference category) and > = 42 weeks [13, 14, 33, 34]. Gestational age was determined by the best clinical estimate, based on ultrasound examination when available, or information on the last menstrual period [35]. Ultrasonography become standard practice in Finland during the late 1980s and early 1990s.

### Chronic disease multimorbidity

Chronic disease multimorbidity was defined as one individual having records of two or more non-communicable chronic diseases (psychiatric or somatic) during the same or subsequent secondary care episode(s). The diseases constituting multimorbidity were selected based on evidence on commonly occurring diseases among preterm and term born adolescents and young adults [21, 24, 25, 36–44]. Multimorbidity was identified using data on both in-patient and out-patient hospital care (referred to in the Nordic context as specialised healthcare), based on diagnostic codes listed in S1 Table in S1 File. First, patient records with two or more of these diagnoses were identified and tagged as indicating chronic disease multimorbidity, with the admission or visit date as the date of onset of multimorbidity. Second, the admissions or visits including only one diagnostic code from S1 Table in S1 File were sorted according to admission date and the date of multimorbidity onset defined as the first time when an individual has accumulated records of two or more chronic diseases. We examined two outcomes: chronic disease multimorbidity in adolescence (age 10–17 years) and in early adulthood (age 18–30 years). These were not mutually exclusive and an individual could have multimorbidity in adolescence as well as in early adulthood.

### Intellectual disabilities and disorders of psychological development, behaviour or personality

Intellectual disabilities and disorders of psychological development, behaviour or personality (which are classified as disorders, rather than diseases, and include, for example, different levels of intellectual disability and autism-spectrum traits) were identified using diagnostic codes provided in S2 Table in S1 File. These were ascertained from each individual’s specialised care records at any time between age 1 year and the end of follow-up. The overlap of chronic disease multimorbidity with these disorders was defined as having a specialised health care record of chronic disease multimorbidity, as well one or more of the diagnostic codes listed in S2 Table in S1 File.

### Covariates

We adjusted the analyses for factors that previous research has shown to be associated with preterm birth [45–50] and which we hypothesised could also be associated with chronic disease multimorbidity in adolescence and early adulthood. These potentially confounding factors were birthweight z-score (calculated using data on birth weight and gestational age, based on Intergrowth Standards [51] as reference values, and modelled as a continuous variable), multiple pregnancy (yes vs. no), any (major or minor) congenital anomalies (yes vs. no), mother’s age (years, continuous), mother’s parity (0, 1, 2, 3, 4, 5+), mother’s smoking during pregnancy (yes vs. no), mother’s socioeconomic position (based on her occupation during pregnancy and categorised as follows: l*ow*: manual workers, pensioners, students and long-term unemployed; *intermediat*e: non-manual workers; *high*: executive employees; *self-employed and unknown*), mother’s diabetes during pregnancy (yes vs. no), mother’s hypertensive disorder during pregnancy (yes vs. no), and year of birth (categories). Mother’s occupation during pregnancy was unknown for 309,882 mothers (32.9%), who were likely to be stay-at-home mothers or students [52]; rather than exclude the children of these mothers from the analyses, we included them in a separate category for mother’s socioeconomic position.

### Statistical analyses

Time at risk of adolescent multimorbidity was defined as time from age 10 to 17 years and time at risk of early adult multimorbidity as time from age 18 to 30 years, up to the first of the following: time of onset of multimorbidity, date of death, date of emigration or the end of follow-up (31^st^ December 2016). Adolescent multimorbidity was modelled conditional on survival to age 10 and early adult multimorbidity conditional on survival to age 18. The analyses were conducted separately for females and males, and for those with intellectual disabilities or disorders of psychological development, behaviour or personality. We estimated hazard ratios (HRs) and 95% confidence intervals (CIs) for both multimorbidity outcomes, across categories of gestational age, using flexible parametric survival models on the cumulative hazard scale, utilising Stata command *stpm2* [53, 54]. With age as the timescale, these models fitted restricted cubic splines with 1 to 6 internal knots (depending on sex and covariates) to model the baseline hazard for each gestational age category. We ran five sets of models for all exposure-outcome pairs: unadjusted models, models adjusted for prenatal exposures (birthweight z-score, multiple pregnancy and congenital anomalies), models adjusted for mother’s characteristics (age, parity, smoking during pregnancy and socioeconomic position), models adjusted for pregnancy disorders (diabetes and hypertension) and multivariable-adjusted models (adjusted for all the above covariates). In addition, we undertook sensitivity analyses adjusted for the year of birth, as a marker for perinatal care. All analyses were conducted using Stata SE 16 (Stata Corporation, College Station, Texas, United States).

## Results

### Study population

Our analyses were based on data from 951,116 individuals were born in Finland during the study period, remained alive and living in Finland at age 10 years and had data available on gestational age, birth weight and covariates (Table 1). During 6,417,903 person-years at risk of multimorbidity in adolescence (median follow-up: 7.9 years), 11,919 individuals (1.3%) had a specialised healthcare record of this outcome (18.6 per 10,000 person-years). During 3,967,419 person-years at risk of early adult multimorbidity (median follow-up: 6.2 years), 15,664 individuals (1.7%) had a specialised healthcare record of this outcome (39.5 per 10,000 person-years). In all, 4,986 individuals (0.5%) died during the follow-up (between the ages of 10 and 30 years).

**Table 1:** Characteristics of study population.

N (%) chronic disease multimorbidity	Gestational age at birth (completed weeks)							
	23–27	28–31	32–33	34–36	37–38	39–41	42+	p
Adolescence (age 10–17 years)	140 (7.0)	207 (5.2)	160 (3.1)	610 (1.8)	2 206 (1.3)	8 076 (1.2)	520 (1.3)	<0.0001
Early adulthood (age 18–30 years)	86 (4.3)	162 (4.1)	141 (2.8)	694 (2.1)	2 856 (1.7)	11 000 (1.6)	725 (1.8)	<0.0001
**Covariates**								
N (%) female	977 (48.9)	1 739 (43.8)	2267 (44.3)	15 718 (46.5)	81 957 (48.4)	353 811 (50.9)	20 768 (50.1)	<0.0001
Median (IQR) birth weight z-score^1^	-0.03 (-1.02 to 0.59)	0.14 (-0.73 to 0.76)	0.14 (-0.54 to 0.72)	0.20 (-0.50 to 0.86)	0.55 (-0.15 to 1.24)	0.71 (-0.04 to 1.38)	0.69 (-0.01 to 1.36)	<0.0001
N (%) multiple pregnancy	378 (18.9)	902 (22.7)	1 320 (25.8)	6 522 (19.3)	11 551 (6.8)	2 848 (0.4)	5 (0.01)	<0.0001
N (%) with any congenital anomalies	255 (12.8)	593 (14.9)	602 (11.8)	3 052 (9.0)	11 824 (7.0)	40 884 (5.9)	2 645 (6.4)	<0.0001
Median (IQR) mother’s age	30 (26 to 34)	30 (25 to 34)	29 (25 to 33)	29 (25 to 33)	29 (26 to 33)	29 (25 to 32)	28 (25 to 32)	<0.0001
N (%) multiparous mother	1 055 (52.8)	1 982 (49.9)	2 505 (48.9)	17 596 (52.0)	104 309 (61.5)	421 545 (60.6)	19 870 (47.9)	<0.0001
N (%) mother smoked during pregnancy	579 (29.0)	1 053 (26.5)	1 220 (23.8)	6 941 (20.5)	31 057 (18.3)	118 483 (17.0)	7 699 (18.6)	<0.0001
N %) mother had low SEP^2^	409 (20.5)	838 (21.1)	1 100 (21.5)	7 029 (20.8)	34 458 (20.3)	143 489 (20.6)	9 461 (22.8)	<0.0001
N (%) hypertension	251 (12.6)	817 (20.6)	1 020 (19.9)	4 944 (14.6)	16 481 (9.7)	35 838 (5.2)	1 495 (3.6)	<0.0001
N (%) diabetes	44 (2.2)	195 (4.9)	329 (6.4)	2 703 (8.0)	13 035 (7.7)	33 021 (4.8)	1 438 (3.5)	<0.0001

^1^ Reference: Intergrowth Standards.

^2^ SEP: socioeconomic position.

### Gestational age and chronic disease multimorbidity

Individuals born preterm were more likely to have multimorbidity in adolescence than those born full-term: multivariable-adjusted HRs for adolescent multimorbidity in females and males, respectively, were 1.29 (95% CI: 1.22 to 1.36) and 1.26 (95% CI: 1.18 to 1.35). The associations of preterm birth with early adult multimorbidity were less marked, with multivariable-adjusted HRs indicating 1.18-fold risk in females (95% CI: 1.12 to 1.24) and 1.10-fold risk in males (95% CI: 1.04 to 1.17).

The associations of categories of gestational age (degrees of prematurity) with chronic disease multimorbidity in adolescence and early adulthood are shown in Tables 2 and 3. We observed a dose-response relationship between increasing prematurity and increasing risks of both multimorbidity outcomes, and again the estimated increased risks were more prominent for adolescent multimorbidity than early adult multimorbidity. For example, the multivariable-adjusted HRs indicate up to 7- and 6-fold risks of adolescent multimorbidity among males and females born at 23–27 weeks, respectively, whereas the corresponding risks for early adult multimorbidity in these groups were approximately 3-fold (Tables 2 and 3). Findings from the sensitivity analyses with additional adjustment for the year of birth were similar our main findings in direction and magnitude (S3 Table in S1 File). Overall, the absolute risks of chronic disease multimorbidity in adolescence and early adulthood in our study population were relatively low, but they were strongly accumulated in prematurely born individuals. The risks of adolescent multimorbidity ranged from 107.1/10,000 person-years in those born at 23–27 weeks to 17.2/10,000 person-years on those born at 39–41 weeks; the corresponding risks of early adult multimorbidity were 106.1/10,000 person years in the very earliest preterm group and 37.9/10,000 person-years among those born at full-term (S4 Table in S1 File).

**Table 2:** Associations of gestational age with chronic disease multimorbidity in adolescence (age 10–17 years) in females and males.

**Gestational age (weeks)**	**HR (95% CI) for chronic disease multimorbidity in adolescence (age 10–17 years)**				
	**Females (n = 477,237)**				
	**Unadjusted**	**Adjusted for prenatal exposures** ^**1**^	**Adjusted for mother’s characteristics** ^**2**^	**Adjusted for pregnancy disorders** ^**3**^	**Multivariable-adjusted** ^**4**^
23–27	6.27 (4.95 to 7.95)	5.88 (4.63 to 7.46)	6.09 (4.81 to 7.72)	6.15 (4.85 to 7.79)	5.65 (4.45 to 7.18)
28–31	4.44 (3.62 to 5.45)	4.18 (3.40 to 5.14)	4.33 (3.53 to 5.32)	4.21 (3.44 to 5.18)	3.88 (3.15 to 4.77)
32–33	2.38 (1.88 to 3.01)	2.31 (1.82 to 2.93)	2.31 (1.82 to 2.92)	2.22 (1.75 to 2.81)	2.10 (1.65 to 2.67)
34–36	1.58 (1.41 to 1.77)	1.56 (1.39 to 1.76)	1.56 (1.40 to 1.75)	1.49 (1.33 to 1.67)	1.46 (1.30 to 1.65)
37–38	1.20 (1.13 to 1.27)	1.19 (1.12 to 1.27)	1.20 (1.13 to 1.27)	1.16 (1.09 to 1.24)	1.16 (1.09 to 1.23)
**39–41**	**1 (ref. cat.)**	**1 (ref. cat.)**	**1 (ref. cat.)**	**1 (ref. cat.)**	**1 (ref. cat.)**
42+	1.03 (0.91 to 1.15)	1.02 (0.91 to 1.15)	1.01 (0.89 to 1.13)	1.03 (0.92 to 1.16)	1.01 (0.90 to 1.14)
**Gestational age (weeks)**	**HR (95% CI) for chronic disease multimorbidity in adolescence (age 10–17 years)**				
	**Males (n = 473,879)**				
	**Unadjusted**	**Adjusted for prenatal exposures** ^1^	**Adjusted for mother’s characteristics** ^2^	**Adjusted for pregnancy disorders** ^3^	**Multivariable-adjusted** ^4^
23–27	7.74 (6.11 to 9.81)	6.94 (5.46 to 8.83)	7.53 (5.94 to 9.55)	7.73 (6.09 to 9.79)	6.85 (5.39 to 8.71)
28–31	5.46 (4.52 to 6.60)	4.79 (3.95 to 5.81)	5.36 (4.44 to 6.48)	5.32 (4.40 to 6.43)	4.64 (3.82 to 5.64)
32–33	3.30 (2.68 to 4.07)	3.02 (2.44 to 3.74)	3.25 (2.63 to 4.01)	3.19 (2.58 to 3.93)	2.91 (2.35 to 3.60)
34–36	1.61 (1.42 to 1.81)	1.51 (1.33 to 1.71)	1.59 (1.41 to 1.79)	1.55 (1.37 to 1.75)	1.45 (1.28 to 1.65)
37–38	1.10 (1.02 to 1.18)	1.08 (1.00 to 1.16)	1.10 (1.02 to 1.18)	1.08 (1.00 to 1.16)	1.06 (0.98 to 1.14)
**39–41**	**1 (ref. cat.)**	**1 (ref. cat.)**	**1 (ref. cat.)**	**1 (ref. cat.)**	**1 (ref. cat.)**
42+	1.13 (0.99 to 1.29)	1.12 80.98 to 1.28)	1.12 (0.98 to 1.27)	1.14 (1.00 to 1.30)	1.11 (0.98 to 1.27)

^1^ Birthweight z-score (continuous), multiple pregnancy (yes vs. no) and congenital anomalies (yes vs. no)

^2^ Mother’s age (years, continuous), parity (0, 1, 2, 3, 4, 5+), smoking during pregnancy (yes vs. no) and socioeconomic position (low, intermediate, high, not known)

^3^ Adjusted for mother’s diabetes (none, gestational diabetes, type 1/type 2 diabetes) and mother’s hypertension during pregnancy (yes vs. no)

^4^ Adjusted for all the above covariates.

**Table 3:** Associations of gestational age with chronic disease multimorbidity in early adulthood (age 18–30 years) in females and males.

**Gestational age (weeks)**	**HR (95% CI) for chronic disease multimorbidity in early adulthood (age 18–30 years)**				
	**Females (n = 324,094)**				
	**Unadjusted**	**Adjusted for prenatal exposures** ^**1**^	**Adjusted for mother’s characteristics** ^**2**^	**Adjusted for pregnancy disorders** ^**3**^	**Multivariable-adjusted** ^**4**^
23–27	3.20 (2.44 to 4.19)	3.11 (2.37 to 4.08)	3.12 (2.38 to 4.08)	3.20 (2.44 to 4.19)	3.04 (2.32 to 3.99)
28–31	2.56 (2.06 to 3.18)	2.48 (1.99 to 3.08)	2.52 (2.03 to 3.12)	2.53 (2.04 to 3.14)	2.40 (1.93 to 2.99)
32–33	1.72 (1.37 to 2.15)	1.70 (1.36 to 2.13)	1.68 (1.34 to 2.10)	1.69 (1.35 to 2.12)	1.62 (1.29 to 2.04)
34–36	1.29 (1.16 to 1.42)	1.28 (1.15 to 1.43)	1.27 (1.15 to 1.41)	1.26 (1.14 to 1.40)	1.24 (1.12 to 1.38)
37–38	1.14 (1.08 to 1.20)	1.14 (1.08 to 1.20)	1.14 (1.08 to 1.20)	1.12 (1.07 to 1.19)	1.12 (1.06 to 1.19)
**39–41**	**1 (ref. cat.)**	**1 (ref. cat.)**	**1 (ref. cat.)**	**1 (ref. cat.)**	**1 (ref. cat.)**
42+	1.05 (0.94 to 1.16)	1.04 (0.94 to 1.15)	1.03 (0.93 to 1.14)	1.05 (0.95 to 1.16)	1.03 (0.93 to 1.14)
**Gestational age (weeks)**	**HR (95% CI) for chronic disease multimorbidity in early adulthood (age 18–30 years)**				
	**Males (n = 322,354)**				
	**Unadjusted**	**Adjusted for prenatal exposures** ^1^	**Adjusted for mother’s characteristics** ^2^	**Adjusted for pregnancy disorders** ^3^	**Multivariable-adjusted** ^4^
23–27	2.79 (1.97 to 3.96)	2.61 (1.83 to 3.70)	2.70 (1.90 to 3.83)	2.79 (1.97 to 3.96)	2.54 (1.78 to 3.60)
28–31	2.88 (2.30 to 3.62)	2.61 (2.07 to 3.29)	2.80 (2.23 to 3.52)	2.86 (2.28 to 2.60)	2.54 (2.01 to 3.20)
32–33	1.76 (1.37 to 2.26)	1.65 (1.28 to 2.13)	1.73 (1.35 to 2.22)	1.73 (1.35 to 2.22)	1.60 (1.24 to 2.06)
34–36	1.29 (1.15 to 1.45)	1.23 (1.09 to 1.39)	1.27 (1.13 to 1.43)	1.27 (1.13 to 1.43)	1.20 (1.06 to 1.35)
37–38	1.06 (0.99 to 1.13)	1.04 (0.97 to 1.11)	1.05 (0.99 to 1.12)	1.05 (0.98 to 1.12)	1.03 (0.96 to 1.10)
**39–41**	**1 (ref. cat.)**	**1 (ref. cat.)**	**1 (ref. cat.)**	**1 (ref. cat.)**	**1 (ref. cat.)**
42+	1.23 (1.10 to 1.38)	1.23 (1.10 to 1.38)	1.22 (1.09 to 1.37)	1.24 (1.10 to 1.38)	1.22 (1.09 to 1.40)

^1^ Birthweight z-score (continuous), multiple pregnancy (yes vs. no) and congenital anomalies (yes vs. no)

^2^ 2 Mother’s age (years, continuous), parity (0, 1, 2, 3, 4, 5+), smoking during pregnancy (yes vs. no) and socioeconomic position (low, intermediate, high, not known)

^3^ Adjusted for mother’s diabetes (none, gestational diabetes, type 1/type 2 diabetes) and mother’s hypertension during pregnancy (yes vs. no)

^4^ Adjusted for all above covariates.

To examine combinations of chronic diseases that typically comprised multimorbidity in our study population, we examined the co-occurrence of individual chronic diseases and their associations with preterm birth (S5 Table in S1 File). Diseases that co-occurred with at least one other chronic disease more often among preterm than full-born individuals were urinary tract cancer, diabetes, schizophrenia, depression, multiple sclerosis, cardiovascular diseases, chronic obstructive pulmonary disease, asthma, coeliac disease and/or dermatitis herpetiformis, rheumatoid diseases, renal disease, epilepsy and cerebral palsy. Compared to full-term born individuals, those born preterm were also more likely to have records of intellectual disabilities and disorders of psychological development, behaviour or personality co-occurring with other chronic diseases.

Unadjusted HRs for the first chronic disease in adolescence or early adulthood are provided in S6 Table in S1 File. These indicate higher risks of any chronic disease in preterm born individuals compared to those born at term, but the estimated risks for the first individual disease were notably smaller than the risks of chronic disease multimorbidity.

### Overlap of chronic disease multimorbidity with intellectual disabilities and disorders of psychological development, behaviour or personality

The co-occurrence of multimorbidity in adolescence and early adulthood with intellectual disabilities and disorders of psychological development, behaviour or personality are shown in Table 4. Among those with intellectual disabilities, preterm birth was associated with considerably increased risks of adolescent multimorbidity (depending on gestational age), but there was no clear evidence for an association of gestational age with early adult multimorbidity in this group. The associations of gestational age with adolescent or early adult multimorbidity among individuals with disorders of psychological development, behavioural syndromes or personality disorders were similar to the main findings, with those born earlier having notably higher risks of these outcomes.

**Table 4:** Co-occurrence of developmental disorders and chronic disease multimorbidity.

**Intellectual disabilities**					
		**Chronic disease multimorbidity in adolescence**		**Chronic disease multimorbidity in early adulthood**	
**Gestational age (weeks)**	**N (%) with intellectual disabilities**	**N (% of individuals with intellectual disabilities)**	**HR**^**1**^ **(95% CI)**	**N (% of individuals with intellectual disabilities)**	**HR**^**1**^ **(95% CI)**
23–27	151 (7.6)	46 (30.5)	23.83 (6.53 to 87.03)	22 (3.5)	1.10 (0.68 to 1.80)
28–31	256 (6.5)	67 (26.2)	6.42 (4.41 to 9.35)	56 (21.9)	1.51 (1.10 to 2.05)
32–33	201 (3.9)	54 (26.9)	4.04 (2.96 to 5.51)	34 (16.9)	0.90 (0.62 to 1.29)
34–36	780 (2.3)	113 (14.5)	1.45 (1.18 to 1.78)	142 (18.2)	0.93 (0.77 to 1.12)
37–38	2 588 (1.5)	344 (13.3)	1.14 (1.01 to 1.30)	599 (23.2)	1.00 (0.91 to 1.10)
39–41	8 589 (1.2)	1 161 (13.5)	**1 (ref. cat.)**	2 380 (27.7)	**1 (ref. cat.)**
42+	583 (1.4)	72 (12.4)	0.78 (0.61 to 0.99)	150 (25.7)	0.92 (0.77 to 1.08)
All	13 148 (1.4)	1 857 (14.1)	-	3 383 (25.7)	-
**Disorders of psychological development**					
		**Chronic disease multimorbidity in adolescence**		**Chronic disease multimorbidity in early adulthood**	
**Gestational age (weeks)**	**N (%) with disorders(s) of psychological development**	**N (% of individuals with disorders(s) of psychological development)**	**HR**^**1**^ **(95% CI)**	**N (% of individuals with disorders(s) of psychological development)**	**HR**^**1**^ **(95% CI)**
23–27	522 (26.1)	79 (15.1)	7.95 (3.84 to 16.47)	36 (6.9)	6.86 (1.82 to 25.85)
28–31	820 (20.7)	89 (10.9)	4.41 (2.78 to 7.00)	64 (7.8)	5.52 (2.57 to 11.84)
32–33	767 (15.0)	69 (9.0)	3.10 (2.20 to 4.37)	38 (5.0)	2.44 (1.43 to 1.96)
34–36	3 181 (9.4)	175 (5.5)	1.60 (1.30 to 1.97)	121 (3.8)	1.45 (1.08 to 1.96)
37–38	11 374 (6.7)	493 (4.3)	1.14 (1.02 to 1.28)	448 (3.9)	1.23 (1.07 to 1.43)
39–41	39 484 (5.7)	1 644 (4.2)	**1 (ref. cat.)**	1 538 (3.9)	**1 (ref. cat.)**
42+	2 688 (6.5)	99 (3.7)	0.81 (0.66 to 1.00)	105 (3.9)	0.87 (0.70 to 1.08)
All	58 836 (6.2)	2 648 (4.5)	-	2 350 (4.0)	-
**Behavioural syndromes**					
		**Chronic disease multimorbidity in adolescence**		**Chronic disease multimorbidity in early adulthood**	
**Gestational age (weeks)**	**N (%) with behavioural syndrome(s)**	**N (% of individuals with behavioural syndrome/s)**	**HR**^1^ **(95% CI)**	**N (% of individuals with behavioural syndrome/s)**	**HR**^1^ **(95% CI)**
23–27	61 (3.1)	13 (21.3)	4.29 (2.41 to 7.64)	12 (19.7)	4.82 (1.82 to 12.75)
28–31	158 (4.0)	20 (12.7)	2.12 (1.32 to 3.40)	30 (19.0)	3.52 (1.86 to 6.65)
32–33	210 (4.1)	23 (11.0)	1.63 (1.06 to 2.52)	24 (11.4)	1.54 (0.90 to 2.64)
34–36	958 (2.9)	90 (9.3)	1.27 (1.01 to 1.59)	120 (12.4)	1.43 (1.08 to 1.88)
37–38	4 151 (2.5)	317 (7.7)	1.00 (0.88 to 1.14)	512 (12.3)	1.21 (1.06 to 1.38)
39–41	16 171 (2.3)	1 278 (7.9)	**1 (ref. cat.)**	1 886 (11.7)	**1 (ref. cat.)**
42+	923 (2.2)	68 (7.4)	0.89 (0.69 to 1.13)	116 (12.6)	0.99 (0.81 to 1.21)
All	22 642 (2.4)	1 809 (8.0)	-	2 700 (11.9)	-
**Personality disorders**					
		**Chronic disease multimorbidity in adolescence**		**Chronic disease multimorbidity in early adulthood**	
**Gestational age (weeks)**	**N (%) personality disorder(s)**	**N (% of individuals with personality disorder7s)**	**HR**^**1**^ **(95% CI)**	**N (% of individuals with personality disorder/s)**	**HR**^**1**^ **(95% CI)**
23–27	128 (6.4)	27 (21.1)	8.18 (3.99 to 16.79)	25 (19.5)	7.44 (3.15 to 17.59)
28–31	306 (7.7)	31 (10.1)	3.09 (1.94 to 4.93)	36 (11.8)	2.69 (1.54 to 1.70)
32–33	382 (7.5)	26 (6.8)	1.68 (1.10 to 2.58)	42 (11.0)	2.21 (1.47 to 3.33)
34–36	2 348 (6.9)	160 (6.8)	1.54 (1.28 to 1.86)	209 (8.9)	1.39 (1.13 to 1.70)
37–38	10 103 (6.0)	575 (5.7)	1.18 (1.06 to 1.30)	833 (8.3)	1.11 (1.01 to 1.23)
39–41	38 923 (5.6)	2 010 (5.2)	**1 (ref. cat.)**	3 362 (8.6)	**1 (ref. cat.)**
42+	2 343 (5.7)	114 (4.9)	0.88 80.73 to 1.07)	197 (8.4)	0.92 (0.79 to 1.07)
All	54 533 (5.7)	2 943 (5.4)	-	4 704 (8.6)	-

^**1**^ Adjusted for sex, birthweight z-score (continuous), multiple pregnancy (yes vs. no), congenital anomalies (yes vs. no), mother’s age (years, continuous), mother’s parity (0, 1, 2, 3, 4, 5+), mother’s smoking during pregnancy (yes vs. no), mother’s socioeconomic position (low, intermediate, high, unknown), mother’s diabetes (none, gestational diabetes, type 1/type 2 diabetes) and mother’s hypertension during pregnancy (yes vs. no).

## Discussion

Our findings, based on nationwide register data in Finland, suggest that compared to individuals born full-term, preterm born individuals have ~1.5- to 7-fold increased risks of chronic disease multimorbidity in adolescence and 1.2- to 3-fold increased risk of multimorbidity in early adulthood, with the estimated risks increasing in a dose-response manner with increasing prematurity. The risk patterns were broadly similar in males and females. There was also some indication that the association of gestational age with increased risk of multimorbidity was particularly prominent among individuals with intellectual disabilities or disorders of psychological development, behaviour or personality.

A growing body of evidence shows that prematurely born individuals have increased risks of individual chronic diseases (including metabolic syndrome [23, 24], ischaemic heart disease [19], asthma [27] and depression [31]) in the early part of the life course. The findings presented here suggest that these risks also translate to increased risks of co-occurrence of multiple diseases among preterm born adolescents and young adults. Multimorbidity is a heterogeneous outcome and it is possible that the multimorbidity in our analyses represents concordant disease combinations (that share aetiology or risk factors) or discordant disease combinations (that co-occur for other reasons). We hypothesise that the associations of preterm birth with multimorbidity in our study population may be driven by the diseases that co-occurred with at least one other chronic disease more often among preterm than full-born individuals: urinary tract cancer, diabetes, schizophrenia, depression, multiple sclerosis, cardiovascular diseases, chronic obstructive pulmonary disease, asthma, coeliac disease, rheumatoid diseases, renal disease, epilepsy and cerebral palsy.

### Potential mechanisms

One possible explanation for the increased risk of multimorbidity across the spectrum of premature gestational ages is that preterm born individuals are exposed to *in utero* and perinatal events that can intervene in organ system development at critical stages (e.g. during neural or vascular site development, nephrogenesis and bone mineralisation) [39]. Examples of *in utero* exposures include mother’s diabetes or hypertensive disorder during pregnancy, which can influence glucose metabolism and insulin sensitivity in the prematurely born individual through shared genetic susceptibility or developmental programming. Perinatal events, e.g. prolonged ventilation, can cause injury and result in inflammation and potentially dysfunctional repair processes, leading to respiratory system disease later in life [39].

Other possibilities include some characteristics of preterm born individuals influencing their risk of multimorbidity. Longitudinal studies of people born very prematurely have reported on a “preterm behavioural phenotype”, characterised by symptoms and disorders associated with inattention, anxiety and social difficulties, as well as low cognitive abilities [28, 55]. Many preterm-born young adults attain lower education [56, 57] and are less likely than their term-born counterparts to have couple relationships, start a family and have children [58]. There is also evidence that preterm birth is associated with certain lifestyle factors, including sedentary behaviour [59, 60]. Multimorbidity could mediate the association of preterm birth with educational achievement or physical activity, or low socioeconomic position, living alone or sedentary lifestyle could be risk factors for multimorbidity among individuals with a preterm-born background. In this context, our findings of consistently increased risks of multimorbidity across the spectrum of premature gestational ages indicate directions for further research into the causal pathways between mental and physical health, and behavioural and socioeconomic characteristics of adolescents and adults born preterm.

### Strengths and limitations

An important strength of our analyses is that we used a large set of nationwide register data, with up to 20 years’ follow-up. These whole-population data captured near-complete information on all births and specialised health care episodes in Finland during the study period, thus minimising the risks of sample selection or loss to follow-up introducing bias to our findings. Furthermore, a dataset of over 950,000 individuals and over 15,000 cases of chronic disease multimorbidity provided sufficient statistical power to precisely estimate the risks of multimorbidity across a wide spectrum of gestational ages, including among extremely prematurely born individuals, who are often under-represented in other types of observational studies.

Limitations of our study derive from using routinely collected register data. The multimorbidity outcomes were ascertained from the nationwide Care Register for Healthcare, which overall has good coverage, with over 95% of individuals and care episodes identifiable by unique personal identity numbers [61]. Validation studies suggest that the accuracy of diagnostic coding for cardiovascular diseases, cancer, psychiatric diseases and autism ranged from 88% to 96% [61]. Although it is thus possible that some multimorbid disease combinations have been incompletely recorded in Care Register for Healthcare, which might have introduced bias in our estimates, we would expect such bias to be unrelated to gestational age or covariates. Also, many chronic diseases (e.g. asthma or well-controlled diabetes) can be managed in primary care and for these diseases, the multimorbidity outcomes in our analyses represent the severe end of the disease spectrum. Consequently, our findings are possibly not generalisable to multimorbidity consisting of less severe combinations of certain diseases. Unfortunately, good quality nationwide primary care data were not available for the time period covered in our investigation and we were thus unable to explore this further. Although our analyses were adjusted for a number of potential confounders, we cannot exclude the possibility that residual confounding from imprecisely measured, unmeasured or unknown confounders has influenced our estimates. For instance, consistently recorded data on mother’s pre-pregnancy weight and pregnancy weight gain were not available for the time period covered in our analyses, and we were unable to adjust our analyses for these.

### Implications

Our findings highlight the long-term health implications of preterm birth. Advances in perinatal care in the past decades mean that increasing proportions of even the smallest prematurely born babies now survive, and as a consequence, increasing numbers of people in paediatric, school, student and occupational health care settings have a preterm-born background. There are currently no clinical guidelines for follow-up of prematurely born individuals beyond childhood. Whilst our observations on their own cannot provide a basis for clinical practice recommendations, they suggest that information on gestational age at birth would be a useful characteristic to include in a medical history when assessing the risk of chronic diseases in adolescent and adult patients. Preterm birth could be one early life exposure that could be used to inform targeted screening or preventive strategies.

## Conclusions

Our findings indicate that earlier gestational age at birth is associated with an increased risk of chronic disease multimorbidity in adolescence and early adulthood. These findings suggest that information on gestational age would be a useful characteristic to include in a medical history when assessing the risk of chronic diseases in adolescent and adult patients.

## Supporting information

S1 File (PDF)

S1 Checklist STROBE statement—checklist of items that should be included in reports of cohort studies. (PDF)
