# Supplementary material for: Preterm birth and the risk of chronic disease multimorbidity in adolescence and early adulthood: A population-based cohort study
Source: PLoS One. 2021 Dec 31;16(12):e0261952. doi: 10.1371/journal.pone.0261952 (PMC8719774; doi:10.1371/journal.pone.0261952)
Supplement: S1 File — (PDF) [file pone.0261952.s001.pdf]

Online Supplement

Preterm birth and the risk of chronic disease multimorbidity in adolescence and early adulthood: A population-based cohort study

Katriina Heikkilä,[1] Anna Pulakka, [1], Johanna Metsälä [1], Suvi Alenius [1,2], Petteri Hovi [1], Mika Gissler [3, 4,5], Sven Sandin [6, 7, 8], Eero Kajantie[1, 9, 10]

[1] Population Health Unit, Finnish Institute for Health and Welfare, Helsinki, Finland

[2] Children's Hospital, University of Helsinki and Helsinki University Hospital, Helsinki, Finland

[3] Information Services Department, Finnish Institute for Health and Welfare, Helsinki, Finland

[4] Academic Primary Health Care Centre, Region Stockholm, Stockholm, Sweden

[5] Department of Molecular Medicine and Surgery, Karolinska Institutet, Stockholm, Sweden

[6] Department of Medical Epidemiology and Biostatistics, Karolinska Institutet, Stockholm, Sweden

[7] Department of Psychiatry, Icahn School of Medicine at Mount Sinai, New York, NY, USA

[8] Seaver Autism Center for Research and Treatment at Mount Sinai, New York, NY, USA

[9] PEDEGO Research Unit, MRC Oulu, Oulu University Hospital and University of Oulu, Oulu, Finland

[10] Department of Clinical and Molecular Medicine, Norwegian University of Science and Technology, Trondheim, Norway

Systematic search for publications on preterm birth and multimorbidity

We searched Pubmed from the inception of the database to 13 Aug 2021, for publications on the association of preterm birth or gestational age at birth with multimorbidity. Our search was conducted without language restrictions. First, search terms identifying preterm birth and multimorbidity were searched in the title and/or abstract: the search terms are detailed in Supplementary Table e1. Second, potentially relevant full-test papers were searched for relevant results. Reference lists of the identified papers were also searched for further relevant publications.

Our search identified 3,853 publications. These reported on studies of specific diseases in preterm and term born individuals, studies of a second disease in populations of preterm born children with a specific index disease (comorbidity) and studies of overall mortality and morbidity among preterm born children and adults. However, we found no longitudinal studies comparing the risks of chronic disease multimorbidity (the risk of one individual developing two or more chronic diseases) in preterm and term born individuals. The studies cited in this article represent a selection of the evidence in this area, rather than an exhaustive corpus of all available publications.

Supplementary Table e1. Search strategy for identifying studies on preterm birth and multimorbidity

Search	TITLE AND/OR ABSTRACT CONTAIN
1	multimorbid* OR multi-morbid*
2	multiple diseas*
3	multiple diagnos*
4	multiple illness*
5	multiple condition*
6	multiple morbid*
7	coexisting diseas*
8	coexisting diagnos*
9	coexisting illness*
10	coexisting condition*
11	coexisting morbid*
12	co-existing diseas*
13	co-existing diagnos*
14	co-existing illness*
15	co-existing condition*
16	co-existing morbid*
17	concurrent diseas*
18	concurrent diagnos*
19	concurrent illness*
20	concurrent condition*
21	concurrent morbid*
22	comorbid diseas*
23	comorbid diagnos*
24	comorbid illness*
25	comorbid condition*
26	co-morbid diseas*
27	co-morbid diagnos*
28	co-morbid illness*
29	co-morbid condition*
30	multiple comorbid*
31	multiple co-morbid*
32	comorbid* OR co-morbid*
	**TO IDENTIFY MULTIMORBIDITY**
33	1 OR 2 OR 3 OR 4 OR 5 OR 6 OR 7 OR 8 OR 9 OR 10 OR 11 OR 12 OR 13 OR 14 OR 15 OR 16 OR 17 OR 18 OR 19 OR 20 OR 21 OR 22 OR 23 OR 24 OR 25 OR 26 OR 27 OR 28 OR 29 OR 30 OR 31 OR 32
	**TITLE OB ABSTRACT CONTAIN**
34	preterm OR pre-term OR premature
35	low birth weight
36	gestational age
	**TO IDENTIFY PRETERM BIRTH**
37	34 OR 35 OR 36
	**TO IDENTIFY MULTIMORBIDITY AND PRETERM BIRTH**
	33 AND 37

Supplementary Table e2. Diagnostic codes used in the *a priori* definition of chronic disease multimorbidity

**Disease**	**ICD-10[1] code(s)**
Cancer	
Lip, oral cavity and pharynx	C00-C14
Digestive organs	C15-C26
Respiratory and intrathoracic organ	C30-C39
Bone and articular cartilage	C40-C42
Melanoma	C43
Mesothelial and soft tissue	C45-C49
Breast	C50
Female genital organs	C51-C58
Male genital organs	C60-C63
Urinary tract	C64-C68
Eye, brain and other parts of central nervous system	C69-C72
Thyroid and other endocrine glands	C73-C75
Ill-defined, other secondary and unspecified sites	C76-C80
Lymphoid, hematopoietic and related tissue	C81-C99
Diabetes	E10-E14
Schizophrenia and schizoaffective disorders	F20-F29
Bipolar disorder	F30-F31
Depression	F32-F33
Multiple sclerosis	G35
Hypertensive heart and/ or renal disease	I11-I13
Ischaemic heart disease	I20-I25
Cerebrovascular disease	I60-I69
Peripheral artery disease	I70, I73.9
Chronic obstructive pulmonary disease	J41-J44
Asthma	J45-J46
Crohn's disease	K50
Ulcerative colitis	K51
Fibrosis and cirrhosis of liver	K74
Coeliac disease and/ or dermatitis herpetiformis	K9, L13
Rheumatoid disease	M05, M06, M08, M09
Renal disease	N00-N19
Epilepsy	G40-G41
Cerebral palsy	G80

[1] International Classification of Diseases, version 10

Supplementary Table e3. Diagnostic codes to identify intellectual disabilities and disorders of psychological development, behaviour or personality

**Disease/disorder**	**ICD-10[1] code(s)**
Typically recorded in childhood or adolescence	
Intellectual disabilities	F70-F79
Disorders of psychological development	F80-F83, F88-F89
Pervasive developmental disorders (incl. autism-spectrum disorders)	F84
Typically recorded in adulthood	
Behavioural syndromes	F50-F59
Disorders of adult behaviour and personality	F60-F69

[1] International Classification of Diseases, version 10

Supplementary Table e4. Associations of gestational age with chronic disease multimorbidity, with additional adjustment for year of birth

Gestational age (weeks)	HR (95% CI) for chronic disease multimorbidity			
	**Adolescence (age 10-17 years)**		**Early adulthood (age 18-30 y ears)**	
	**Females (n=477 237)**	**Males (n=473 879)**	**Females (n=324 094)**	**Males (n=322 354)**
23-27	5.40 (4.25 to 3.86)	6.63 (5.22 to 8.44)	3.01 (2.30 to 3.95)	2.52 (1.78 to 3.59)
28-31	3.87 (3.15 to 4.76)	4.60 (3.79 to 5.58)	2.39 (1.92 to 2.97)	2.52 (2.00 to 3.17)
32-33	2.13 (1.67 to 2.70)	2.90 (2.34 to 3.60)	1.62 (1.30 to 2.04)	1.59 (1.23 to 2.05)
34-36	1.48 (1.31 to 1.60)	1.46 (1.29 to 1.66)	1.24 (1.12 to 1.38)	1.19 (1.06 to 1.35)
37-38	1.15 (1.08 to 1.23)	1.05 (0.98 to 1.13)	1.12 (1.06 to 1.18)	1.03 (0.96 to 1.10)
**39-41**	**1 (ref. cat.)**	**1 (ref. cat.)**	**1 (ref. cat.)**	**1 (ref. cat.)**
42+	1.01 (0.90 to 1.14)	1.12 (0.98 to 1.28)	1.03 (0.93 to 1.15)	1.22 (1.09 to 1.37)

[1] Birthweight z-score (continuous), multiple pregnancy (yes vs. no), congenital malformations (yes vs. no), mother's age (years, continuous), mother's parity (0, 1, 2, 3, 4, 5+), mother's smoking during pregnancy (yes vs. no), mother's socioeconomic position (low, intermediate, high, not known), mother's diabetes (none, gestational diabetes, type 1/type 2 diabetes), mother's hypertension during pregnancy (yes vs. no) and year of birth (1987 to 2006).

Supplementary Table e5. Absolute risks of chronic disease multimorbidity, by gestational age

**Gestational age (weeks)**	**Chronic disease multimorbidity in adolescence (per 10 000 person-years)**	**in early adulthood (per 10 000 person-years)**
23-27	107.1	106.1
28-31	76.2	93.8
32-33	44.4	61.4
34-36	25.8	46.4
37-38	19.2	40.7
39-41	17.2	37.9
42+	18.9	43.3
**Overall**	**18.6**	**39.5**

Supplementary Table e6. Co-occurring diseases and their associations with preterm birth

**Disease**	**N (%) individuals**	**N (%) individuals with this and at least one another disease**	**Unadjusted OR (95% CI) for having this and at least one another, preterm vs. full-term born[1]**
**Chronic diseases**			
Cancer			
Lip, oral cavity and pharynx	23 (≤0.1)	11 (47.8)	2.16 (0.61 to 7.66)
Digestive organs	37 (≤0.1)	19 (51.4)	0.69 (0.20 to 2.42)
Respiratory and intrathoracic organ	12 (≤0.1)	6 (50.0)	0.65 (0.08 to 5.55)
Bone and articular cartilage	70 (≤0.1)	35 (50.0)	1.22 (0.57 to 2.62)
Melanoma	30 (≤0.1)	14 (46.7)	0.88 (0.25 to 3.17)
Mesothelial and soft tissue	78 (≤0.1)	40 (51.3)	1.08 (0.53 to 2.21)
Breast	27 (≤0.1)	10 (37.0)	0.41 (0.05 to 3.24)
Female genital organs	83 (≤0.1)	24 (28.9)	0.31 (0.07 to 1.32)
Male genital organs	55 (≤0.1)	36 (65.5)	1.85 (0.91 to 3.77)
Urinary tract	48 (≤0.1)	23 (47.9)	**2.43 (1.02 to 5.77)**
Eye, brain and other parts of central nervous system	237 (≤0.1)	121 (51.1)	1.16 (0.77 to 1.74)
Thyroid and other endocrine glands	111 (≤0.1)	56 (50.5)	1.49 (0.84 to 2.65)
Ill-defined, other secondary and unspecified sites	54 (≤0.1)	49 (90.7)	1.08 (0.56 to 2.08)
Lymphoid, hematopoietic and related tissue	612 (≤0.1)	262 (42.8)	1.27 (0.96 to 1.67)
Diabetes	12 527 (1.3)	3 366 (26.9)	**1.27 (1.18 to 1.38)**
Schizophrenia and schizoaffective disorders	11 614 (1.2)	7 274 (62.6)	**1.08 (1.02 to 1.14)**
Bipolar disorder	6 094 (0.6)	4 738 (77.8)	0.99 (0.93 to 1.06)
Depression	66 873 (7.0)	17 126 (25.6)	**1.13 (1.09 to 1.17)**
Multiple sclerosis	645 (≤0.1)	213 (33.1)	**1.43 (1.06 to 1.92)**
Hypertensive heart and/ or renal disease	78 (≤0.1)	39 (50.0)	**2.11 (1.10 to 4.05)**
Ischaemic heart disease	725 (≤0.1)	272 (37.5)	**1.69 (1.31 to 2.19)**
Cerebrovascular disease	1 398 (0.15)	580 (41.5)	**1.26 (1.05 to 1.52)**
Peripheral artery disease	141 (≤0.1)	54 (38.3)	1.48 (0.82 to 2.68)
Chronic obstructive pulmonary disease	503 (≤0.1)	326 (64.8)	**1.40 (1.10 to 1.78)**
Asthma	57 196 (6.0)	8 610 (15.1)	**1.31 (1.25 to 1.38)**
Crohn’s disease	3 270 (≤0.1)	1 912 (58.5)	1.07 (0.96 to 1.19)
Ulcerative colitis	5 227 (0.6)	2 291 (43.8)	1.07 (0.97 to 1.18)
Fibrosis and cirrhosis of liver	49 (≤0.1)	37 (75.5)	1.43 (0.70 to 2.90)
Coeliac disease and/or dermatitis herpetiformis	1 770 (0.2)	1 195 (67.5)	**1.24 (1.09 to 1.42)**
Rheumatoid disease	6 513 (0.7)	1 634 (25.1)	**1.24 (1.11 to 1.39)**
Renal disease	353 (≤0.1)	111 (31.0)	**2.11 (1.44 to 3.10)**
Epilepsy	5 510 (0.6)	2 497 (45.3)	**1.82 (1.67 to 1.98)**
Cerebral palsy	2 444 (0.3)	1 176 (48.1)	**4.29 (3.82 to 4.83)**
**Intellectual disabilities and disorders of psychological development, behaviour or personality**			
Intellectual disabilities	13 148 (1.4)	4 648 (35.4)	**1.31 (1.23 to 1.40)**
Disorders of psychological development (incl. autism-spectrum disorders)	58 836 (6.2)	4 422 (7.5)	**1.65 (1.54 to 1.75)**
Behavioural syndromes	22 642 (2.4)	4 018 (17.8)	**1.17 (1.09 to 1.25)**
Disorders of adult behaviour and personality	54 533 (5.8)	6945 (12.7)	**1.17 (1.11 to 1.24)**

[1]Preterm and early term: 23-38 weeks; full-term: 39-41 weeks.

Supplementary Table e7. Associations of preterm birth with the first chronic disease[1]

Gestational age (weeks)	Unadjusted HR (95% CI) for chronic disease multimorbidity			
	**Adolescence (age 10-17 years)**		**Early adulthood (age 18-30 years)**	
	**Females (n=477,237)**	**Males (n=473,879)**	**Females (n=324,094)**	**Males (n=322,354)**
23-27	2.17 (1.94 to 2.42)	2.54 (2.28 to 2.83)	1.64 (1.44 to 1.87)	1.87 (1.64 to 2.14)
28-31	2.28 (2.10 to 2.46)	2.65 (2.47 to 2.85)	1.82 (1.66 to 1.99)	2.07 (1.91 to 2.25)
32-33	1.67 (1.55 to 1.80)	1.71 (1.59 to 1.85)	1.39 (1.27 to 1.52)	1.50 (1.38 to 1.64)
34-36	1.26 (1.21 to 1.30)	1.28 (1.24 to 1.33)	1.15 (1.11 to 1.20)	1.20 (1.15 to 1.25)
37-38	1.10 (1.08 to 1.12)	1.07 (1.05 to 1.10)	1.09 (1.07 to 1.12)	1.05 (1.03 to 1.07)
**39-41**	**1 (ref. cat.)**	**1 (ref. cat.)**	**1 (ref. cat.)**	**1 (ref. cat.)**
42+	1.06 (1.02 to 1.09)	1.04 (1.00 to 1.08)	1.10 (1.09 to 1.14)	1.07 (1.02 to 1.11)

[1] First disease is defined as the first occurrence of any disease listed in Table e1, at age 10-17 years or 18-30 years.
