# Supplementary material for: Preterm birth and the risk of chronic disease multimorbidity in adolescence and early adulthood: A population-based cohort study
Source: PLoS One. 2021 Dec 31;16(12):e0261952. doi: 10.1371/journal.pone.0261952 (PMC8719774; doi:10.1371/journal.pone.0261952)
Supplement: S1 Checklist — (PDF) [file pone.0261952.s002.pdf]

Preterm birth and the risk of chronic disease multimorbidity in adolescence and early adulthood: A population-based cohort study

Katriina Heikkilä,[1] Anna Pulakka, [1], Johanna Metsälä [1], Suvi Alenius [1,2], Petteri Hovi [1], Mika Gissler [3, 4,5], Sven Sandin [6, 7, 8], Eero Kajantie[1, 9, 10]

1 Population Health Unit, Finnish Institute for Health and Welfare, Helsinki, Finland

2 Children's Hospital, University of Helsinki and Helsinki University Hospital, Helsinki, Finland

3 Information Services Department, Finnish Institute for Health and Welfare, Helsinki, Finland

4 Academic Primary Health Care Centre, Region Stockholm, Stockholm, Sweden

5 Department of Molecular Medicine and Surgery, Karolinska Institutet, Stockholm, Sweden

6 Department of Medical Epidemiology and Biostatistics, Karolinska Institutet, Stockholm, Sweden

7 Department of Psychiatry, Icahn School of Medicine at Mount Sinai, New York, NY, USA

8 Seaver Autism Center for Research and Treatment at Mount Sinai, New York, NY, USA

9 PEDEGO Research Unit, MRC Oulu, Oulu University Hospital and University of Oulu, Oulu, Finland

10 Department of Clinical and Molecular Medicine, Norwegian University of Science and Technology, Trondheim, Norway

STROBE Statement—Checklist of items that should be included in reports of ***cohort studies***

	Item No	Recommendation	Reported on page no.
**Title and abstract**	1	(*a*) Indicate the study's design with a commonly used term in the title or the abstract	1-3
		(*b*) Provide in the abstract an informative and balanced summary of what was done and what was found	2-3
**Introduction**			
Background/rationale	2	Explain the scientific background and rationale for the investigation being reported	4-5
Objectives	3	State specific objectives, including any prespecified hypotheses	4-5
**Methods**			
Study design	4	Present key elements of study design early in the paper	5-6
Setting	5	Describe the setting, locations, and relevant dates, including periods of recruitment, exposure, follow-up, and data collection	5-6
Participants	6	(*a*) Give the eligibility criteria, and the sources and methods of selection of participants. Describe methods of follow-up	6
		(*b*) For matched studies, give matching criteria and number of exposed and unexposed	NA
Variables	7	Clearly define all outcomes, exposures, predictors, potential confounders, and effect modifiers. Give diagnostic criteria, if applicable	6-8
Data sources/ measurement	8*	For each variable of interest, give sources of data and details of methods of assessment (measurement). Describe comparability of assessment methods if there is more than one group	6-8
Bias	9	Describe any efforts to address potential sources of bias	8-9
Study size	10	Explain how the study size was arrived at	6, 9
Quantitative variables	11	Explain how quantitative variables were handled in the analyses. If applicable, describe which groupings were chosen and why	6-8
Statistical methods	12	(*a*) Describe all statistical methods, including those used to control for confounding	8-9
		(*b*) Describe any methods used to examine subgroups and interactions	8-9
		(*c*) Explain how missing data were addressed	6
		(*d*) If applicable, explain how loss to follow-up was addressed	NA
		(*e*) Describe any sensitivity analyses	9
**Results**			
Participants	13*	(a) Report numbers of individuals at each stage of study—eg numbers potentially eligible, examined for eligibility, confirmed eligible, included in the study, completing follow-up, and analysed	9-10
		(b) Give reasons for non-participation at each stage	NA
		(c) Consider use of a flow diagram	-
Descriptive data	14*	(a) Give characteristics of study participants (eg demographic, clinical, social) and information on exposures and potential confounders	9-10, Table 1
		(b) Indicate number of participants with missing data for each variable of interest	8

		(c) Summarise follow-up time (eg, average and total amount)	9-10
Outcome data	15*	Report numbers of outcome events or summary measures over time	9-10
Main results	16	(*a*) Give unadjusted estimates and, if applicable, confounder-adjusted estimates and their precision (eg, 95% confidence interval). Make clear which confounders were adjusted for and why they were included	10,12 Tables 2-4
		(*b*) Report category boundaries when continuous variables were categorized	NA
		(*c*) If relevant, consider translating estimates of relative risk into absolute risk for a meaningful time period	9-10, Online Supplement, Table e4
Other analyses	17	Report other analyses done—eg analyses of subgroups and interactions, and sensitivity analyses	13, Online Supplement, Tables e5 and e6
**Discussion**			
Key results	18	Summarise key results with reference to study objectives	18
Limitations	19	Discuss limitations of the study, taking into account sources of potential bias or imprecision. Discuss both direction and magnitude of any potential bias	12
Interpretation	20	Give a cautious overall interpretation of results considering objectives, limitations, multiplicity of analyses, results from similar studies, and other relevant evidence	19
Generalisability	21	Discuss the generalisability (external validity) of the study results	20
**Other information**			
Funding	22	Give the source of funding and the role of the funders for the present study and, if applicable, for the original study on which the present article is based	22

*Give information separately for exposed and unexposed groups.

Note: An Explanation and Elaboration article discusses each checklist item and gives methodological background and published examples of transparent reporting. The STROBE checklist is best used in conjunction with this article (freely available on the Web sites of PLoS Medicine at http://www.plosmedicine.org/, Annals of Internal Medicine at http://www.annals.org/, and Epidemiology at http://www.epidem.com/). Information on the STROBE Initiative is available at http://www.strobe-statement.org.
